# Single-Cell Genomics for Virology

**DOI:** 10.3390/v8050123

**Published:** 2016-05-04

**Authors:** Angela Ciuffi, Sylvie Rato, Amalio Telenti

**Affiliations:** 1 Institute of Microbiology, University Hospital Center and University of Lausanne, Bugnon 48, CH-1011 Lausanne, Switzerland; Sylvie.Ferreira-Rato@chuv.ch; 2 The J. Craig Venter Institute, 4120 Capricorn Lane, La Jolla, CA 92037, USA; atelenti@jcvi.org

**Keywords:** single cell, heterogeneity, genomics, transcriptomics, proteomics, sequencing, RNA-Seq, virus, microfluidics, cell-to-cell variability

## Abstract

Single-cell sequencing technologies, *i.e.*, single cell analysis followed by deep sequencing investigate cellular heterogeneity in many biological settings. It was only in the past year that single-cell sequencing analyses has been applied in the field of virology, providing new ways to explore viral diversity and cell response to viral infection, which are summarized in the present review.

## 1. Introduction

Cell population analysis is an essential tool to investigate cellular biology and response to infection. However, cell populations consist of a mixture of cells that may differ in their identity (cell type/subpopulation/lineage), in their state/process (cell cycle, circadian rhythm), or just due to stochastic variation. Rare cells and subsets, potentially relevant for some specific phenotypes, might be missed by cell population analyses. The steps to address cell heterogeneity include: (i) single cell separation and isolation (such as micromanipulation, fluorescence-activated cell sorting (FACS), microdroplets, microfluidics); and (ii) sensitive analysis that can accommodate and process the limited amount of biological material (deep sequencing (DNA-Seq, ATAC-Seq, RNA-Seq), FACS, single cell mass cytometry (CyTOF), time-lapse microscopy) (recently reviewed in [1,2,3]). Single-cell sequencing studies performed at the genome-wide or transcriptome-wide levels have already proven useful for many application fields, including cancer, immunology, embryology, and microbiology [1].

Heterogeneity in cell population occurs in cells thought to share an identical DNA. However, there is increasing interest in characterizing the levels of somatic variation at DNA level. Indeed, cells within one organism all derived initially from the same zygote cell, thus, from one single source of genomic information. However, with subsequent divisions and under different microenvironmental influences, cells accumulate genomic (somatic) mutations. An example to illustrate the importance of somatic heterogeneity is cancer. Here, analysis may reveal the existence of rare transformed individual cells, differences in driver mutations, and diverse resistance mechanisms in response to treatment [4,5,6,7,8,9]. There is also a great interest in the identification of somatic structural variations in normal tissues—in particular, in the brain [10].

Transcriptome analysis of single cells is now broadly used as well—and significant information in this review will refer to this. The observation of single cell heterogeneity in response to viral infection is well established, and already the focus of sophisticated approaches [11]. Publications on single cell studies followed by deep sequencing only took off about one year ago. The aim of this review is to provide an overview of single cell studies (summarized in Table 1) while focusing more on single-cell sequencing studies published to date in the field of virology, aiming at exploring viral diversity as well as individual cell variability in response to viral replication.

## 2. The Origins of Single Cell Heterogeneity at the Transcriptome Level

As mentioned above, cell-to-cell transcription variability depends on the cell identity itself at first. The pool of T cells is a great example of such heterogeneity as it is composed of many different cell subtypes and subsets [6,9,12,13,14,15]. Additional layers of variability have been shown to originate in the cell population context, *i.e.*, the local cell density and the cell position in the population [11], in the cell cycle [16], and in the transcription stochasticity. Recently, it has been shown that noise due to stochastic variation of transcription was quite low when considering cytoplasmic transcripts [17,18,19]. Indeed, cell-to-cell variation at the transcriptional level was present in the nucleus but not in the cytoplasm, suggesting that transcripts were retained in the nucleus, thereby buffering transcript variation in the cytoplasm. The mechanisms involved in nuclear retention of transcripts remain to be elucidated but may involve epitranscriptomic regulation, *i.e.*, chemical modifications of RNA molecules such as m^6^A methylation [20]. The presence of nuclear transcript retention may also provide an explanation for the low correlation between transcript levels and protein levels, in contrast to ribosome-associated transcript levels and protein levels. Therefore, it would be interesting to determine the level of correlation between cytoplasmic transcripts and proteins.

Cellular heterogeneity will, thus, affect virus replication from the initial infection of the cell to viral release, and thus also affect the clinical outcome of the viral infection. The impact of cell-to-cell variation on the viral life cycle can be investigated from the point of view of the virus, *i.e.*, by analyzing viral sequences, or from the point of view of the cell, *i.e.*, by analyzing the cellular transcriptome.

## 3. Virus-Based Analyses

### 3.1. The Impact of Cellular Heterogeneity on Viral Replication (Expression and Yield)

**Figure 1:**
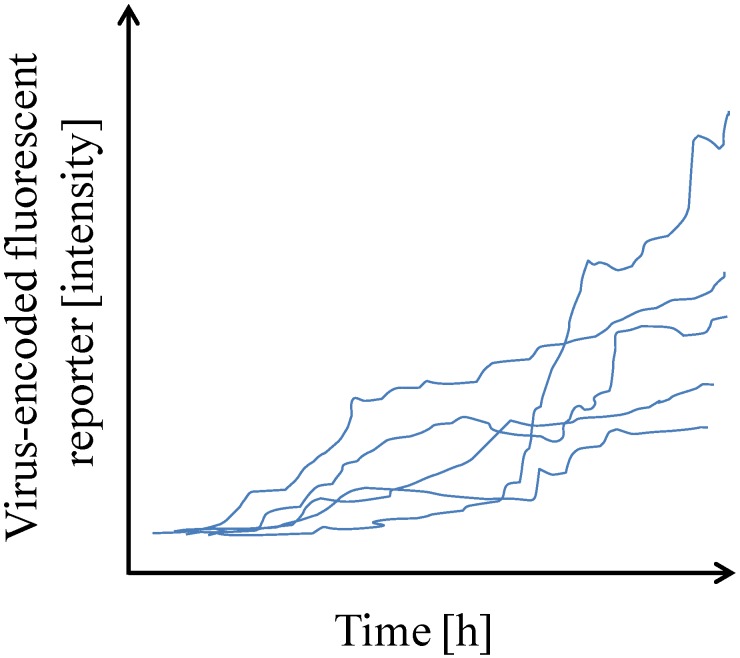
Single cells display cell-to-cell variability in gene expression. Graph plot showing the expression of a virus-encoded fluorescent reporter over time post-infection as quantified by time-lapse microscopy. (Adapted from [21]).

As mentioned above, single cell analysis has to deal with single cell isolation and processing of limited amount of sample. Single cell isolation was easier to overcome than subsequent analysis. Thus, initial studies mostly investigated one viral-encoded fluorescent reporter gene over time in single cells by FACS or by time-lapse microscopy [21,22] (Table 1). These studies led to the first appreciations of cell-to-cell variability in human immunodeficiency virus (HIV) gene expression and helped determining that infected cells usually contained one integrated HIV copy (Figure 1).

Although delayed as being technically more challenging, single-cell proteomics is also moving forward for single cell phenotyping, growing from the simultaneous detection of up to 18 proteins by FACS to more than 40 proteins by CyTOF, and providing a valuable tool to start characterizing variation of cellular protein expression at the single cell level [23,24]. A large majority of single cell studies investigated cellular heterogeneity at the level of viral gene expression and infectious virion progeny production using specific reverse transcription polymerase chain reaction (RT-PCR) and plaque assays (Table 1).

#### 3.1.1. Vesicular Stomatitis Virus (VSV)

**Figure 2:**
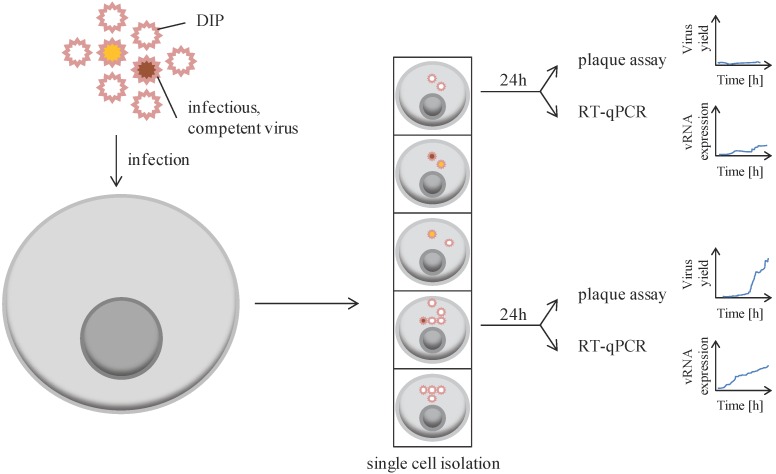
Single cells display cell-to-cell variability in infectious viral progeny. Cells are infected at a determined multiplicity of infection, usually represented by a virus aggregate containing infectious (colored) and defective particles (empty, defective interfering particles, DIP). The genomic sequence of infectious, competent viruses may vary among viral particles, represented here by a yellow and a red particle. After infection, cells are separated and grown for 24 h. Cells are then collected for assessing specific gene expression (usually viral RNA) by RT-qPCR, while supernatants are collected to perform plaque assay for infectious titer determination.

VSV, containing a non-segmented, single-stranded RNA genome of negative polarity, was used as a first model to explore cellular heterogeneity at the level of production of infectious particles (Figure 2). Initial studies showed that 24 h post-infection at a multiplicity of infection (MOI) of 5, the virus yield of infectious particle progeny was affected by cell cycle, with G2M phase associated with a higher number of plaque forming units (PFU) than the S phase. More importantly, the yield ranged from 50 to 8000 PFU per single cell [40]. This cell-to-cell variability in virion progeny production was investigated for many other viruses (as listed in Table 1). A few of these studies are detailed below.

Akpninar *et al.* analyzed the impact of defective interfering particles (DIP) aggregated to infectious particles for overall infection success [43]. For this, BHK-21 cells were co-infected for 24 h with a fixed MOI (MOI = 30) of VSV competent particles and variable amounts of VSV-DIP. The success of infection was followed by titration of infectious virion progeny (plaque assay) or by detection of a virally-encoded fluorescent reporter (time-lapse microscopy) (Figure 2). The number of infectious particles (PFU) produced by infected cells was reduced when higher amounts of DIP were used during initial co-infection. Similarly, the expression level of the virus-encoded fluorescent reporter was also reduced with high amounts of DIP. All together, these data suggest that VSV-DIP interfere with successful and productive infection of replication-competent VSV, probably due to particle competition for intracellular resources necessary for viral genome replication, expression, and production. This system also allowed following viral gene expression over time within the same cell, thereby informing on cell-to-cell variability, as well as the kinetics of viral protein synthesis and expression. Finally, cell density may also impact single cell behavior as an isolated single cell might behave differentially than an individual cell in context of a population. Indeed, DIP interfere more with isolated single cells compared to single cells in a dense population, as assessed by viral reporter expression and virus yield.

Combe *et al.* analyzed cellular heterogeneity in the outcome of VSV infection [44]. For this purpose, they infected Baby Hamster Kidney (BHK)-21 cells with VSV particles that were previously sequenced to know the input viral genomic diversity, identifying 197 single-nucleotide polymorphisms (SNP, parental variants). Infected cells were then separated by micromanipulation and incubated for 24 h, thereby allowing two rounds of virus generation. Supernatants were used to quantify infectious virion progeny by plaque assay, followed by deep sequencing to explore genetic diversity. Results, derived from a total of 90 infected cells and 881 plaques (7–10 plaques per infected cell), first identified a total of 532 SNP, 36 originated in the viral stock and 496 newly arising SNP, corresponding to a mutation rate of 2.8 × 10^−5^ mutations per nucleotide per cell infection (or on average 5.51 new SNP identified in 7–10 plaques), and allowing a rapid gain of genetic diversity. A second observation relied in the presence of multiple parental variants in many infected cells consistent with virus co-infection. Indeed, data were consistent with the hypothesis that one infectious unit was composed of an aggregate of virions, in which at least one was infectious and replication competent while the others were mostly defective (DIP). This observation suggests that cells are mostly co-infected by multiple viral variants, enabling a rapid generation of genetic diversity in the virion progeny.

#### 3.1.2. Hepatitis C Virus (HCV)

McWilliam, Leitch, and McLauchlan investigated HCV, a positive single-stranded RNA virus. In particular, they analyzed the viral diversity of HCV replicon quasi-species by RT-qPCR and vRNA deep-sequencing in individual cells [29]. They determined that on average, one single cell contained 113 copies of replicon RNA (ranging from 84 to 160 copies). Furthermore, analysis of viral variants highlighted a large dominance of wild type (wt) sequence, although minor variants were also identified.

#### 3.1.3. Hepatitis B Virus (HBV)

Zhang *et al.* investigated HBV infection and quantified at single cell level the amount of intracellular viral nucleic acids, which are cytoplasmic vRNA and vDNA, as well as nuclear covalently-closed circular DNAs (cccDNA) [27]. *In situ* hybridization assay on liver biopsies of chronic hepatitis B infection was able to detect HBV cccDNA in patients’ cells, even after one year of patient treatment, suggesting the high-level resistance and persistence of this viral genomic form. Furthermore, this latent stage of infection also co-occurred with the absence of detection of the HBV surface antigen (HBsAg). All together, these data highlighted a specific temporal pattern of HBsAg expression, virion production, or cccDNA detection, which co-occur with productive or latent phase of HBV life cycle.

#### 3.1.4. Influenza A Virus (IAV)

Heldt *et al.* investigated cell-to-cell variability in IAV infection, which contains eight negative single-stranded genomic segments [36]. For this, they infected MDCK cells, isolated the infected cells by serial dilution and analyzed intracellular viral RNA (vRNA) of single cells by RT-qPCR as well as virion progeny by plaque assay 12 h post-infection. Key findings of this study revealed high cellular heterogeneity due to both intrinsic and extrinsic noise origins, *i.e.*, stochastic variation and use of cellular resources (biochemical reactions necessary for intracellular viral life cycle progression), respectively. Indeed, they first showed variability in vRNA expression levels between infected cells as well as differences in the copy number of the various vRNA segments within the same infected cell. These vRNA expression differences eventually led to up to 3-log differences in release of virion progeny, with single cells releasing from 1 to 970 PFU. Furthermore, many infected cells were unable to release viral particles, either due to the presence of defective interfering particles (DIP), due to failure to achieve endosomal fusion, or due to vRNA degradation (genomic segment loss).

## 4. Cell-Based Analyses

### 4.1. The Impact of Cellular Heterogeneity on Virus-Induced Cellular Immune Response

Tsioris *et al.* investigated the humoral immune response of West Nile Virus (WNV)-infected patient cells [46]. The authors collected blood samples from infected patients with recent or post-convalescent WNV infections, isolated B cell subpopulations and processed them using a single cell analysis approach (microengraving) aiming at capturing, sequencing, and characterizing WNV-specific antibodies. At the same time, analysis of the B cell repertoire (*i.e.*, heavy chain sequences) was carried out. Data revealed that WNV-specific cells persisted over time, during patients’ post-convalescence and that the humoral response was not predictive of the WNV infection severity. The authors also identified four novel WNV neutralizing antibodies (NAb). A similar analysis on the humoral immune response for Dengue Virus (DENV) was carried out by Cox *et al.* using a FACS-based approach to capture DENV-specific memory B cells, followed by differentiation and secreted antibody characterization on one hand and by single cell sequencing of antibody sequence on the other hand [25]. This method allowed for quantification of Dengue-specific memory B cells and Ab functional characterization.

### 4.2. The Impact of Viral Infection on Cellular Transcriptome

Luna *et al.* investigated HCV and the role of miR-122 binding to the viral RNA genome in favoring HCV replication in hepatoma cells [28]. The use of single cell analysis by FACS of two bidirectional fluorescent reporters was carried out to confirm a “sponge” effect of miR-122. Indeed, in absence of HCV, miR-122 binds to cellular mRNA targets, thereby promoting their degradation and, thus, reducing related encoded proteins. In presence of HCV, that binds and thus sequesters miR-122, the free pool of miR-122 is reduced, thereby leading to a de-repression of miR-122 target cellular mRNAs and to an increase of the corresponding encoded proteins. To summarize, miR-122 acts as a translational regulator of a specific subset of cellular mRNAs and can be blocked by HCV.

### 4.3. The Impact of Cellular Heterogeneity on Viral Infection Outcome

Wu *et al.* investigated the cellular heterogeneity of virally-induced tumors [34]. To achieve this, they analyzed in detail the whole transcriptome of HeLa S3 cells, a HPV18-infected cell line derived from a cervical cancer. They developed an automated pipeline, named micro-well full-length mRNA amplification and library construction system (MIRALCS), to isolate >500 single cells, perform the reverse transcription, amplify the cDNA, and prepare the library for deep-sequencing (single ends/50 nucleotides for 37 individual cells and paired-ends/150 nucleotides for eight individual cells). They observed a cell-to-cell variation in the number of total transcripts detected, ranging from 67,000 to 233,000 transcript copies per cell. Cellular heterogeneity was also highlighted at the level of splicing variation, including virus-host fusion transcripts. Finally, expression of viral E6 and E7 oncogenes was co-incident with a cluster of 281 cellular genes, potentially involved in virus-mediated cellular transformation.

Ciuffi *et al.* investigated cell heterogeneity by studying the differential permissiveness of primary CD4+ T cells to HIV infection [33]. The authors established a single cell RNA-Seq approach with a dedicated bioinformatic pipeline to identify biomarkers of HIV permissiveness [47]. More than 80 cells from each cell donor, *i.e.*, displaying the high permissive or low permissive phenotype, were analyzed using a microfluidics to isolate single cells and RNA-Seq. Transcripts displaying a differential and bimodal expression were considered as candidate biomarkers and were further tested.

Similarly, Martin-Gayo *et al.* studied cell heterogeneity of a subset of dendritic cells (DCs) from Elite Controller patients (EC) previously described to have a more efficient immune response against HIV-1 infection [48,49]. The authors single-cell sorted DCs (85 cells) that were previously challenged with HIV-1 and performed a whole transcriptome analysis. Single-cell RNA-Seq revealed three different transcriptional patterns that differed mainly on the expression of interferon stimulated genes (ISGs), cytokines, cytokine receptors, and co-stimulatory molecules. Focusing on one of the transcription profiles, the authors were able to identify specific markers that characterize a highly-functional subset of DCs with improved abilities to induce T cell proliferation.

## 5. Discussion

**Figure 3:**
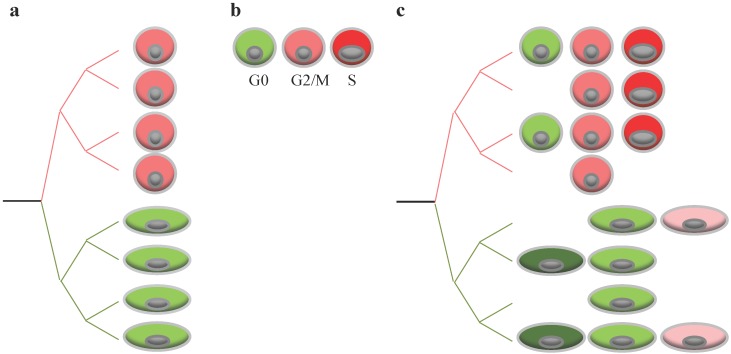
Single cell analysis and impact of cell-to-cell variability on virus replication**.** (**a**) Cell population may contain multiple cell subsets, displaying high (red) and low (green) susceptibility to viral replication; (**b**) cell population may contain cells in different states (differentiation, cell cycle), affecting their susceptibility to viral infection. For example, cells in the S phase might display high levels of susceptibility to viral infection (red) while cells in G0 a low susceptibility to infection (green); and (**c**) complexity of cell population when cellular heterogeneity is a mixture of cells of multiple subsets, in multiple states, and in different proportions.

The single cell technology has proven to be a valuable tool to explore DNA, RNA, or proteins in one single cell at a time. To achieve that goal, this technology had to deal with ways to isolate and capture cells, and to accommodate limited amounts of biological material. Although most studies currently focus on one type of omic data only (DNA, RNA, epigenome), the technology is still evolving, already allowing gathering multiple omic data (e.g., DNA and RNA) from one same single cell [50]. Finally, recent effort is being devoted to increase the throughput of single cell analysis in order to collect data from a higher number of individual cells. Thus, single cell analyses allows for re-examination of key viral features of replication in the context of simple models, as represented by a single cell (Figure 3a,b). Investigating the impact of virus replication on specific cell subsets or specific cell states should help reconstitute the viral phenotype observed at the cell population (Figure 3c). Single cell transcriptome analysis and single cell proteomics should help identify a cell-specific signature, informing on both cell lineage and cell state in an unbiased way.

## Figures and Tables

**Table 1:** Single cell analysis studies.

Virus ^a^	Cells ^b^	Single Cell Isolation	Biological Sample Analyzed ^c^	Method	Reference
SV40 (RV, MHV, DENV)	HeLa	-	Immunofluorescence	Microscopy and imaging	Snijder *et al.* [11]
DENV	B cells from DENV seropositive patients	FACS sorting	Cellular RNA	Deep-sequencing	Cox *et al.* [25]
FMDV	BHK-21	Micromanipulation	vRNA	RT-PCR	Huang *et al.* [26]
HBV	Primary hepatocytes	Microscopy	vRNA, vDNA, cccDNA	*In Situ* Hybridization	Zhang *et al.* [27]
HCV	Huh-7.5 (hepatoma cell line)	FACS	Virus-encoded BFP and RFP reporters	FACS	Luna *et al.* [28]
HCV	Huh-7 (hepatoma cell line)	Micromanipulation	vRNA	Deep-sequencing, RT-qPCR	McWilliam Leitch and McLauchlan [29]
HIV	Jurkat (human T cell line)	Micromanipulation	Virus-encoded GFP	Time-lapse microscopy, FACS	Weinberger *et al.* [22]
HIV	Primary human CD4+ T cells (from blood)	Micromanipulation	Virus-encoded GFP	Time-lapse microscopy	Razooky *et al.* [21]
HIV	Primary human CD4+ T cells (from infected patients)	Microdissection	vDNA	PCR-Sequencing	Suspène and Meyerhans [30]
HIV	Primary human CD4+ T cells (from infected patients)	FACS sorting	vDNA, vRNA	Deep-sequencing	Josefsson *et al.* [31]
HIV	Primary human CD4+ T cells	Microfluidics (Fluidigm)	RNA	Deep-sequencing	Ciuffi *et al.* [32,33]
HPV	HeLaS3	MIRALCS pipeline	Cellular RNA and vRNA	RNA-Seq	Wu *et al.* [34]
HTLV	Cytotoxic T lymphocytes from Adult-T-Leukemia (ATL) patients	FACS sorting	Cellular RNA (T-Cell Receptor repertoire)	Deep-sequencing	Tanaka *et al.* [35]
IAV	MDCK	Micromanipulation	vRNA, virion progeny	RT-qPCR, plaque assay	Heldt *et al.* [36]; Laske *et al.* [37]
Poliovirus	HeLaS3	FACS sorting	Virion progeny, vRNA	Plaque assay, RT-qPCR	Schulte and Andino [38]; Schulte *et al.* [39]
VSV	BHK-21	FACS sorting	Virion progeny	Plaque assay	Zhu *et al.* [40]
VSV	BHK-21	Micromanipulation	Virion progeny	Plaque assay	Timm and Yin [41]
VSV	BHK-21	Micromanipulation	Virion progeny (Virion-associated vRNA)	Deep-sequencing	Timm *et al.* [42]
VSV	BHK-21	Micromanipulation	Virus-encoded GFP, virion progeny	Time-lapse microscopy, plaque assay	Akpinar *et al.* [43]
VSV	BHK-21	Micromanipulation	Virion progeny (Virion-associated vRNA)	Deep-sequencing, plaque assay	Combe *et al.* [44]
VZV	Primary human T cells (from tonsils)	Mass Cytometry (CyTOF)	Set of 40 protein markers	Mass Cytometry (CyTOF)	Sen *et al.* [23,45]
WNV	Primary B cells (from infected patients)	Microengraving pipeline	Cellular RNA, Antibodies	Deep-sequencing, neutralization assay	Tsioris *et al.* [46]

^a^ DENV Dengue Virus; FMDV Foot-and-Mouth Disease virus; HBV Hepatitis B Virus; HCV Hepatitis C Virus; HIV Human Immunodeficiency Virus type 1; HPV Human Papilloma Virus; HTLV Human T-cell Leukemia virus; IAV Influenza A Virus; MHV Mouse Hepatitis Virus; RV Rotavirus; SV40 Simian Virus 40; VSV Vesicular Stomatitis Virus; VZV Varicella Zoster Virus; WNV West Nile Virus; ^b^ BHK-21 Baby Hamster Kidney; MDCK Madine-Darby Canine Kidney; ^c^ GFP green fluorescent protein; vRNA viral RNA (intracellular); cccDNA covalently closed circular DNA.
